# Osteosynthesis of non-displaced femoral neck fractures in the elderly population using the femoral neck system (FNS): short-term clinical and radiological outcomes

**DOI:** 10.1186/s13018-021-02622-z

**Published:** 2021-08-04

**Authors:** Oscar Vazquez, Axel Gamulin, Didier Hannouche, Wilson Belaieff

**Affiliations:** Division of Orthopaedic and Trauma Surgery, Department of Surgery, University Hospitals of Geneva, 4 Rue Gabrielle-Perret-Gentil, CH-1211, Geneva 14, Switzerland

**Keywords:** Femoral neck fracture, Elderly population, Osteosynthesis, Orthogeriatrics, FNS, Femoral neck system, DHS, Dynamic hip screw, Triple screws

## Abstract

**Background:**

Femoral neck fractures (FNF) are frequent in the elderly population, and surgical management is indicated in the vast majority of cases. Osteosynthesis is an alternative to arthroplasty for non-displaced FNF. Triple screw construct (TS) and the dynamic hip screw system (DHS) are considered gold standards for osteosynthesis. The newly available femoral neck system (FNS) currently lacks evidence as to whether it is a valid alternative to TS and DHS. The purpose of this study was to evaluate the short-term clinical and radiological outcomes after non-displaced (Garden I and II) FNF osteosynthesis using TS, DHS, and FNS.

**Methods:**

All the patients of the author’s institution aged ≥ 75 years with a non-displaced (Garden I and II) FNF eligible for osteosynthesis between November 2015 and December 2019 were included in this single-center retrospective non-randomized study. Patients were treated with either TS, DHS, or FNS depending on the surgeon’s preference. Clinical data (age, gender, ASA score, duration of surgery, need for blood transfusion and number of packed red blood cells transfused, surgical site complications, length of stay, discharge location, postoperative medical complications and readmission within 30 days, and mortality within 3 months) were extracted from the patients’ charts. The radiological analysis assessed the fracture classification, fracture impaction, and proximal femur shortening at 3 and 6 months using the institutional imaging software.

**Results:**

Baseline characteristics in the TS (*n* = 32), DHS (*n* = 16), and FNS (*n* = 15) groups were similar with respect to age (mean 85 years), gender (female to male ratio 4:1), and ASA score. There were no significant differences across the groups for the need for blood transfusion, surgical site complications, length of stay, postoperative medical complications and readmission within 30 days, discharge location, and mortality within 3 months. The duration of surgery was significantly lower in the FNS group (43.3 vs 68.8 min; *p* < 0.001). The radiological assessment found similar impaction (5.2 mm ± 4.8) and shortening (8.6 mm ± 8.2) in all groups that did not seem to progress after 3 months.

**Conclusion:**

The FNS appears to be a valid alternative implant for FNF osteosynthesis and is associated with a shorter operative time than TS and DHS. Short-term clinical and radiological outcomes of FNS are similar to TS and DHS implants. Further long-term multicenter randomized studies are however necessary to confirm these first results.

## Background

Femoral neck fractures (FNF) are common in the elderly population and are associated with significant morbidity and mortality [1]. It is estimated that at the age of 80, the risk of developing a fracture of the proximal femur is approximately 20% for women and 10% for men [2]. Surgical management of FNF is indicated in the vast majority of elderly patients, and the indication to fix or to replace the fracture depends on fracture displacement and patient selection [3]. Elderly patients presenting with non-displaced (Garden I and II) FNF with a posterior tilt of > 20° and those with displaced (Garden III and IV) FNF will preferentially benefit from hip arthroplasty [4–6]. Stable non-displaced (Garden I and II) FNF may be managed with either hip arthroplasty or osteosynthesis [7], and the optimal treatment for such fractures is still subject to debate [6, 8]. Osteosynthesis is associated with higher complication rates than arthroplasty, such as non-union (20 to 35%) [9], avascular necrosis of the femoral head (23%) [10] fracture impaction, and consecutive abductor insufficiency (27%) [11]. This results in revision rates up to three times higher than with arthroplasty ranging from 10 to 49% [12, 13]. However, osteosynthesis has several advantages in this population, including a shorter operative time, less physiological stress [14], and reduced blood loss and risk of infection [15]. Immediate full weight-bearing is usually allowed, and low revision rates have been reported after non-displaced FNF fixation. The most commonly used implants for non-displaced FNF fixation are the triple screw construct (TS) and the dynamic hip screw system (DHS) [16]. The TS construct provides good torsional stability, preserves blood flow to the femoral head, and can be performed through a minimally invasive approach [17]. This technique, however, provides limited resistance to vertical shear forces at the fracture site [18]. The DHS design provides better resistance to vertical shear forces but requires a larger incision for implantation and bears a higher risk of avascular necrosis of the femoral head [19]. A large international, multicenter, randomized controlled trial failed to show any difference in terms of outcomes between TS and DHS [20].

The femoral neck system (FNS) is a novel device available since 2018 (https://www.jnjmedicaldevices.com/sites/default/files/user_uploaded_assets/pdf_assets/2019-11/118534-190712%20DSUS_EM%20). This system combines a short lateral plate that holds one or two locking screws with a fixed-angle tunnel allowing a diverging blade and screw construct to recoil through the plate. Biomechanical studies of this novel implant report high resistance to shear, torsion, and compression forces [21]. However, there is no in vivo literature available to date regarding this new device. Therefore, the aim of the present study was to assess the short-term radiological and clinical outcomes after non-displaced (Garden I and II) FNF using the FNS, in comparison with TS and DHS.

## Methods

**Fig. 1:**
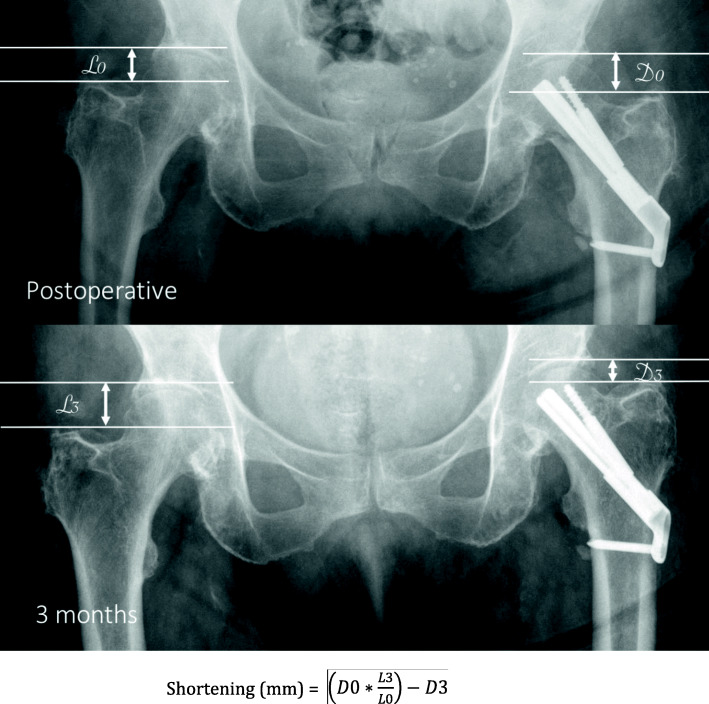
Calculation of proximal femur shortening with DHS, TS, and FNS. Postoperative and 3-month X-ray

**Fig. 2:**
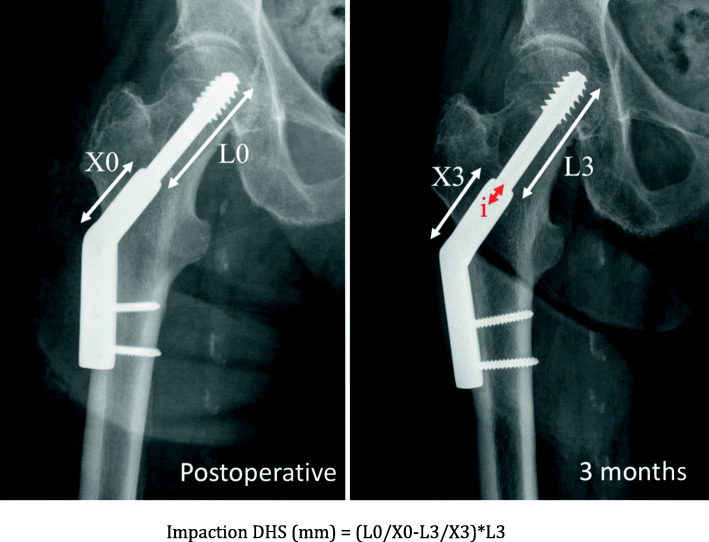
Calculation of impaction with DHS implant. Postoperative and 3-month X-ray

**Fig. 3:**
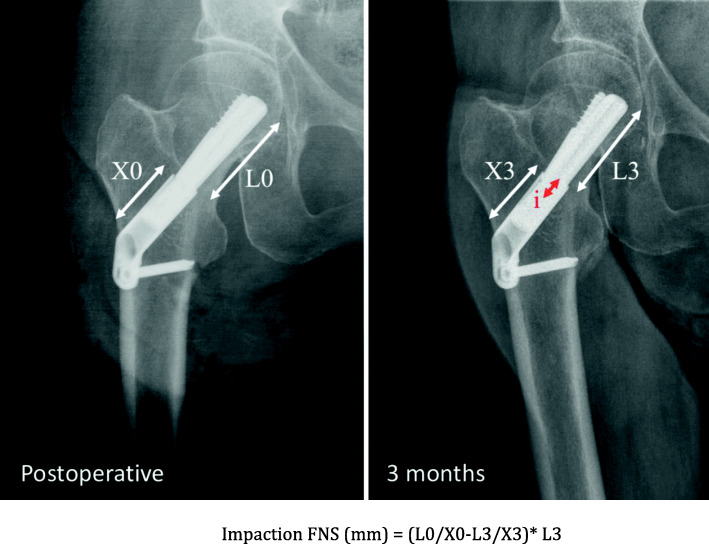
Calculation of impaction with FNS implant. Postoperative and 3-month X-ray

**Fig. 4:**
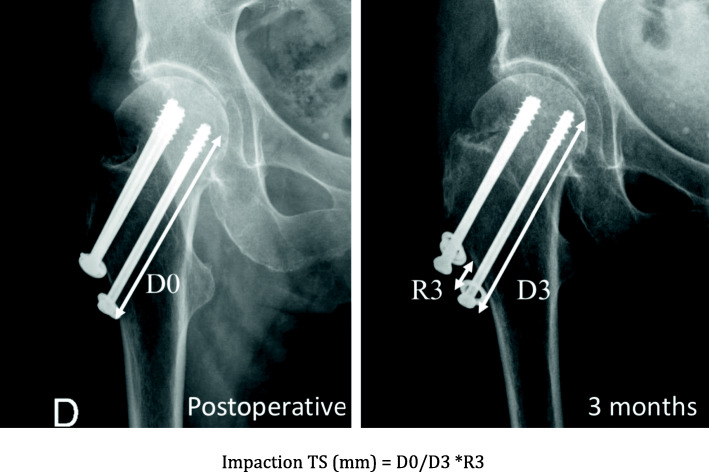
Calculation of impaction with TS implant. Postoperative and 3-month X-ray

All patients > 75 years admitted between November 2015 and December 2019 with a non-displaced FNF (Garden I and II, posterior tilt < 20°) and eligible for fixation were included in this retrospective single-center non-randomized study. Patients are cared for by a multidisciplinary comanaged clinical pathway, and no major changes were brought to this setup during the study period, besides rotation of the team’s members within the division. Surgery was performed by four residents supervised by a consultant, and by 7 staff surgeons mitigating the effect of advanced trauma surgeon experience on operative time. All data were collected in the fracture registry available in our institution. All patients were allowed to fully bear weight with crutches or a walking frame after surgery. The following clinical data were extracted from patients’ charts: age, gender, ASA score, duration of surgery, need for blood transfusion and number of packed red blood cells transfused, surgical site complications (local or implant infection, peri-implant fracture, wound dehiscence), length of stay (LOS), discharge location, postoperative medical complications and readmission within 30 days, and mortality within 3 months. Standardized radiographic images were obtained preoperatively, postoperatively, and at 3 and 6 months after surgery, as per standard institutional protocol. Radiological analysis of proximal femur shortening was assessed according to Zhang et al. [22] and Felton et al. [11] (Fig. 1). Impaction was calculated by analysis of the displacement of the screw normalized to the length of the barrel for DHS and FNS (Figs. 2 and 3) and by measurement of the recoil of the screws normalized to screw length for TS (Fig. 4). All measurements were performed using a dedicated web-based open-source PACS workstation DICOM viewer (Weasis medical viewer, available on https://nroduit.github.io/en/). All data were anonymized and stored in a computerized database.

Categorical variables were expressed as proportion, and for continuous variables, mean, standard deviations, and ranges were reported. Patients’ characteristics and outcomes were compared between the groups (TS, DHS, FNS) using the chi-square test or Fisher exact test for qualitative parameters, and linear regression model for quantitative parameters, except for length of stay, which was compared between the groups using a Kruskal-Wallis test. Finally, the overall effect of surgery on radiographic outcomes was assessed using mixed effects linear regression models with random effect on patients and fixed effects on surgery and time, with no interaction term between fixed effects (i.e., we hypothesized that the effect of surgery, if any, was the same at 3 and 6 months). Statistical significance was assessed at the two-sided 0.05 level for all analyses. All analyses were performed using R version 4.0.2. This study was carried out in accordance with the Chart of Helsinki. This study followed the recommendations of the STROBE guidelines.

## Results

### Clinical outcomes

**Fig. 5:**
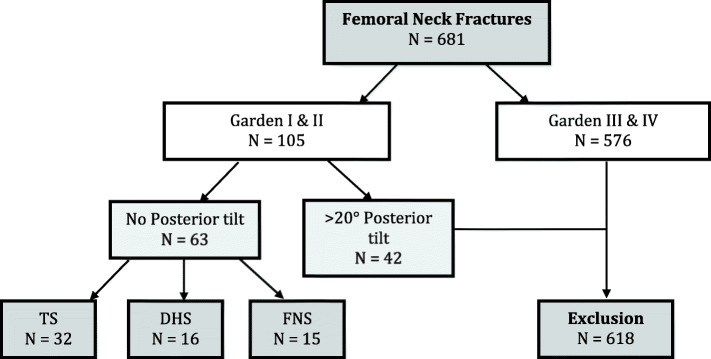
Flow chart showing the distribution of fractures by Fig implants

We analyzed 681 patients with FNF, of which 576 were displaced and 105 were non-displaced. Among non-displaced FNF, 42 were candidates for arthroplasty due to a posterior tilt > 20°, and 63 for osteosynthesis (Fig. 5). The latter were classified as Garden I and II in 54 and 9 cases, respectively. Fracture fixation was performed with TS, DHS, and FNS in 32, 16, and 15 patients, respectively. The 3 groups were comparable for age (TS 85, DHS 81; FNS 87, *p* = 0.48), gender (TS: female/male 28:4; DHS: female/male 10:6; FNS: female/male 13:2; *p* = 0.128), and ASA score (*p* = 0.726). The mean age for the TS, DHS, and FNS groups was 85.0, 83.4, and 86.1, respectively (Table 1).

**Table 1:** Baseline characteristics

Characteristics	Modality	All (*n* = 63)	TS (*n* = 32)	DHS (*n* = 16)	FNS (*n* = 15)	*P* value
Age (years)	Mean (sd)	84.9 (6.4)	85 (6.6)	83.4 (7.3)	86.1 (4.6)	0.48
	Median (IQR)	85 (79–90)	85 (79.8–90)	81 (77.8–88.8)	87 (85–88.5)	
	Range	75–100	75–100	76–98	75–92	
	Missing	0	0	0	0	
Gender	Female (*n* (%))	51 (81)	28 (88)	10 (62)	13 (86)	0.128
	Male (*n* (%))	12 (19)	4 (12)	6 (36)	2 (13)	
ASA	2 (*n* (%))	20 (32)	11 (34)	6 (38)	3 (20)	0.726
	3 (*n* (%))	35 (56)	18 (56)	8 (50)	9 (60)	
	4 (*n* (%))	8 (12)	3 (10)	2 (12)	3 (20)	

*TS* triple screw, *DHS* dynamic hip screw system, *FNS* femoral neck system

The male to female ratio was 1:4. The average operative time for TS, DHS, and FNS was 66.9, 70.7, and 43.3 min, respectively. The FNS group showed a significantly shorter intraoperative time than TS and DHS (p < 0.001) (Table 2). The average LOS for TS, DHS, and FNS was 12.2 days (range 5–30), 12.4 (range 8–27), and 10.3 (range 5–25), respectively. The shorter LOS in the FNS group did not reach statistical significance. At least one blood transfusion was necessary in 3 patients in the TS group, none in the DHS group, and 1 in the FNS group. One single 30-day surgical postoperative complication was noted in the TS group warranting revision for infection. No early surgical revisions due to technical error or failure of the implant were noted. Thirty-seven percent of all patients developed at least one medical complication within 30 days after surgery. These were noted in 14 (43%), 4 (24%), and 5 (26%) patients of the TS, DHS, and FNS groups, respectively, without reaching statistical significance. Overall, patient discharge was possible to a rehabilitation center, nursing facility, and home in 63%, 25%, and 11% cases, respectively. Transfer to a rehabilitation center was noted in 20, 11, and 9 patients in the TS, DHS, and FNS groups, respectively. One case from the FNS group required hospital readmission at 30 days, due to an acute onset pulmonary edema. The overall 3-month mortality was 8%, divided in 3 patients for the TS group, 2 patients for the DHS group, and 0 patients for the FNS group.

**Table 2:** Clinical outcomes

Characteristics	Modality	All (*n* = 63)	TS (*n* = 32)	DHS (*n* = 16)	FNS (*n* = 15)	*P* value
Duration of surgery (min)	Mean (sd)	62.2 (23.6)	66.9 (25.4)	70.7 (20)	43.3 (10.1)	**< 0.001**
	Median (IQR)	55 (46–76)	63.5 (50–80)	66 (53–83)	42 (37.5–52.5)	
	Range	20–128	20–128	50–114	28–63	
	Missing	0	0	0	0	
Blood transfusion (*n* (%))	0	59 (94)	29 (90)	16 (100)	14 (93)	0.466
	1	2 (3.2)	2 (6.3)	–	–	
	2	2 (3.2)	1 (3.1)	–	1 (6.7)	
Surgical site complications (*n* (%))	0	62 (98)	31 (97)	16 (100)	15 (100)	1
	1	1 (1.6)	1 (3,1)	–	–	
Length of stay (days)	Mean (sd)	11.8 (5.9)	12.2 (6.2)	12.4 (5.3)	10.3 (6)	0.131
	Median (IQR)	10 (8–13.5)	10 (8–15)	10.5 (9–13.2)	8 (6–10.5)	
	Range	5–30	5–30	8–27	5–25	
	Missing	0	0	0	0	
Discharge location (*n* (%))	Rehabilitation	40 (63)	20 (63)	11 (69)	9 (60)	0.509
	Home	7 (11)	2 (6)	3 (19)	2 (13)	
	Nursing home	16 (2)	10 (31)	2 (13)	4 (27)	
Medical complication (30 days) (*n*(%))	0	40 (63)	18 (56)	12 (75)	10 (67)	0.441
	1	7 (11)	4 (13)	1 (6)	2 (13)	
	2	11 (17)	7 (22)	2 (13)	2 (13)	
	3	2 (3)	1 (3)	1 (6)	–	
	4	2 (3)	1 (3)	–	1 (6.7)	
	5	1 (2)	1 (3)	–	–	
Readmissions (30 days) (*n* (%))	1	1 (2)	–	–	1 (6.7)	0.238
	0	62 (98)	32 (100)	16 (100)	14 (93)	
Death (3 months)	1	5	3	2	–	0.585
	0	58	29	14	15	

Surgical site complications include local or implant infection, peri-implant fracture, and wound dehiscence. Medical complications include heart failure, respiratory failure, urinary tract infection, and pulmonary embolism

*TS* triple screw, *DHS* dynamic hip screw system, *FNS* femoral neck system

### Radiographic outcomes

In our study population, 15 patients (24%) did not have any documented radiological follow-up, 13 (20%) had 1 3-month X-ray, and 35 (56%) had both 3- and 6-month X-ray. Reasons for incomplete radiological follow-up were due to living abroad (4), death (5), refusal (1), and inability (18) to attend postoperative consultations.

Radiographic analysis showed an average of 5.2 ± 4.8 mm fracture impaction at 3 months when compared to immediate postoperative X-rays. The TS, DHS, and FNS groups showed a similar mean impaction of 5.0 ± 4.5, 5.4 ± 6, and 5.5mm ± 4.4, respectively. In mixed linear regression model, the type of surgery had no statistically significant effect on impaction (*p* = 0.872) and impaction was not statistically significantly different between 3 and 6 months (*p* = 0.979). Analysis of proximal femur shortening at 3 months showed an average 8.6-mm ascension of the trochanter with respect to the acetabular roof. The TS, DHS, and FNS groups had an 8.4 ± 7.0, 8.3 ± 11.9, and 9.3mm ± 6.0 shortening, respectively. In the mixed linear regression model, the type of surgery had no statistically significant effect on shortening (*p* = 0.982), and shortening was not statistically significantly different between 3 and 6 months (*p* = 0.218) (Table 3).

**Table 3:** Impaction and shortening at 3 and 6 months

Characteristics	Modality	All (*n* = 63)	TS (*n* = 32)	DHS (*n* = 16)	FNS (*n* = 15)	*P* value
Impaction						
3 months	Mean (sd)	5.2 (4.8)	5 (4.5)	5.4 (6)	5.5 (4.4)	
	Median (IQR)	4.9 (1.7–7.2)	4.9 (2.5–6.6)	2.6 (1.4–7.7)	5.4 (1.8–9.4)	
	Range	0–21.1	0–18	0.2–21.1	0.2–13.1	
	Missing	15	10	3	2	
6 months	Mean (sd)	5.4 (5.2)	5.4 (4.8)	5.6 (5.9)	5 (6.7)	
	Median (IQR)	4.7 (2.6–6.9)	4.7 (3.2–5.9)	4.5 (2–8.1)	7.3 (3.4–8.9)	
	Range	− 4.8–19.6	0–19.6	− 2.9–18.7	− 4.8–10.1	
	Missing	30	13	6	11	
Global						0.872
Δ 3–6 months						0.979
Shortening						
3 months	Mean (sd)	8.6 (8.2)	8.4 (7.0)	8.3 (11.9)	9.3 (6.0)	
	Median (IQR)	8.3 (2.4–12.1)	8.6 (4.3–12.6)	8.4 (0.3–11.4)	8.3 (5.7–13.3)	
	Range	− 6.2–42	− 6.2–22.3	− 4.1–42	1.1–22	
	Missing	15	10	3	2	
6 months	Mean (sd)	7.0 (9.0)	8.1 (10.3)	4.9 (7.9)	6.7 (4.0)	
	Median (IQR)	6 (1.2–10.5)	5 (2.5–11.4)	6.7 (− 2.3–12.4)	7.8 (4.9–9.6)	
	Range	− 5.8–38.9	− 3.9–38.9	− 5.8–13.6	1.2–10.1	
	Missing	30	13	6	11	
Global						0.982
Δ 3–6 months						0.218

*TS* triple screw, *DHS* dynamic hip screw system, *FNS* femoral neck system

## Discussion

Fixation options for non-displaced FNF in the elderly population are associated with several advantages such as reduction in operative time and reduced blood loss in comparison with hip replacement surgery. To date, no implant has been proven superior to another for FNF osteosynthesis [20, 23]. The FNS is a novel implant designed to address the low resistance to shearing forces of TS and allow better rotational stability than the DHS. To the best of the authors’ knowledge, no studies to date have evaluated the clinical and radiological outcomes of the FNS.

Operative time is an important factor when considering surgery in frail patients. Longer surgeries are associated with higher blood loss, longer anesthesia, higher infection rates, and overall higher postoperative complication rates [24]. Our study found a significantly shorter operative time in the FNS group, with an average of 43.3 min (vs. 66.9 min). Eleven different surgeons (seven staff surgeons and four residents under supervision) have implanted the FNS, and none had experience with this implant before the start of the study. Also, none of them has implanted the FNS more than 2 times. This finding highlights the relative simplicity of the FNS technique, despite a theoretical learning curve for this novel implant.

Anemia is a negative predictive factor for survival after hip fracture surgery [25]. As for TS and to a lesser extent the DHS, the FNS is designed to be implanted through a minimally invasive approach using a single lateral small-sized 2–3-cm incision. Our study failed to identify any significant postoperative differences in the need for transfusion between the groups, despite a theoretical larger incision and soft tissue trauma associated with DHS. Moreover, it is possible that preoperative bleeding from the fracture site, regardless of the time to surgery, maybe a confounding factor in the occurrence of postoperative anemia. Our study did however not directly assess absolute hemoglobin values pre- and postoperatively.

In light of the budgetary constraints and the economic burden of fragility fractures, LOS is a much looked upon variable to measure the quality of care and efficiency. The average in-hospital stay in the study’s population was 11.8 days. This is less than the LOS of patients with arthroplasties for FNF (12.8 days, authors’ institutional *Elderly Proximal Femoral Fracture Register*, unpublished data). Our study suggests a trend toward a shorter stay in the FNS group (10.4 days) without reaching statistical significance. This potential shorter stay associated with FNS may be beneficial in terms of resources available on the wards and lowering the burden on healthcare professionals in charge of this highly dependent patient population.

Femoral neck fractures frequently result in an important loss of function despite appropriate rehabilitation. One of the reasons for this is the occurrence of a limp, with or without leg length discrepancy. Functional shortening of the femoral neck due to fracture impaction may result in the loss of the abductors’ moment of force on the greater trochanter, resulting in weakness, pain, and patient dissatisfaction [11]. In this study, TS, DHS, and FNS were associated with some degree of radiologic femoral neck impaction and proximal femur shortening during follow-up. However, none of the implants was significantly more prone to do so. From that perspective, despite our small group of patients, the FNS seems to be at least as effective as other implants in maintaining a stable fracture reduction. It is also important to acknowledge that, in contrast to TS and DHS, some technical inaccuracies in choice of size and positioning of the implants due to a lack of experience with this new implant may have negatively influenced our outcomes. Despite this, no patient in the FNS group needed revision surgery due to technical error.

This study has several limitations. First, it may be subject to methodological bias due to its retrospective and observational design. Secondly, clinical and radiological outcomes were reported with a low number of patients. This is due to the fact that non-displaced FNF are fairly uncommon as they represent 15% of all FNF (authors’ institutional *Elderly Proximal Femoral Fracture Register*, unpublished data). A larger scale study, possibly multicenter, would be desirable to confirm our preliminary results. Also, the FNS has only been recently made available in the authors’ institution, providing fewer cases than TS or DHS to include in the analysis. Third, 24% of the patients were lost to follow-up in the current series, but this is in the range of most studies on FNFs in elderly patients. In a multicenter randomized trial on 201 patients comparing cemented and uncemented hemiarthroplasty, and Moerman and al. found a similar 24% rate of loss to follow-up, which was mainly due to a high number of patients with cognitive disorders and high age of the participators who were unable to adhere to postoperative follow-up visits [26]. Fourth, our study was focused on short-term outcomes, and hypothesized that most adverse events correlated with the use of the device (FNS, DHS, or triple screws), would occur within 6 months after surgery. A long-term follow-up would be useful in detecting delayed complications such as AVN, as well as hardware-related trochanteric pain. Fifth, we acknowledge that fracture healing evaluation with a CT scan would have been more accurate to detect non-union, but this is not performed routinely in our department, neither in most studies published on this topic. In a recent study evaluating the outcomes of intracapsular non-displaced FNFs in 244 patients treated with cannulated screws, mechanical failure of fixation was identified on standard X-rays, which is considered as the standard of care in this elderly population [27]. Lastly, this study was not designed to evaluate the clinical correlation between the secondary displacement of the fracture and clinical or gait abnormalities.

## Conclusion

To the best of the authors’ knowledge, this is the first study to evaluate the clinical and radiological outcomes of the FNS. In comparison with TS and DHS, the use of the FNS is potentially associated with a significantly shorter operative time and appears to be as effective as TS and DHS in preventing early secondary fracture displacement. The FNS appears to be a valid alternative to other FNF fixation techniques in non-displaced FNF in the elderly population. Further high-quality, large volume, long-term multicenter randomized studies are necessary to confirm these first results.

## Data Availability

The detailed datasets and materials of this study are available from the corresponding author through emails upon request.
